# Obesity

**Published:** 1902-02-08

**Authors:** 


### The Hospital

A Journal of

The Medical Sciences and Hospital Administration.

Vol. XXXI.—No. 802.] Edited by Sir Henry Burdett, K.C.B., and Solomon C. Smith, M.D., M.R.C.P. [February 8, 1902.

### OBESITY.

It cannot be said that the discussion on obesity at the last meeting of the Medical Society was very enlightening; it was, however, useful, insisting as it did upon certain principles which must be borne in mind if we are to be successful in arranging a course of treatment for this sometimes very troublesome and even serious condition. It was pointed out that in essence the accumulation of fat in the body depends on too much fat or fat-forming food being taken into the body, and on too little being burned off. But it is essential to bear in mind, as was indicated by Sir Lauder Brunton, that fat may be formed from various kinds of food, even though they may themselves contain no fat, such as albuminates and carbohydrates, so that, although it is clear enough that the tendency to lay on fat is determined to a considerable extent by the nature of the food, it would be taking a very one-sided view of the question to regard this as everything. There is evidently in some people a "vice of metabolism," a constitutional condition which makes almost every kind of food "run to fat," as the saying is, so that Dr. Burney Yeo's classification of cases of obesity into two types—those that are easy to treat and those that are difficult to treat—simple as it sounds, becomes one of considerable practical importance; the obesity in the one class of cases being due to some habit of life or of feeding on the removal of which the fat melts away, while in the other so great is the tendency to store up food in the form of fat rather than to use it in the production of energy or in the repair of waste that the stress of any treatment which aims at curing obesity by diminishing food or by increasing exercise is apt to fall upon the more important tissues rather than on that which we wish to remove and thus to cause debility and exhaustion rather than absorption of the adiposity. This is the cause of most of the real difficulty in the management of these cases. Still, even in the worst and most "constitutional" of cases treatment can do much. The whole of the obesity which is present is not the direct result of the constitutional tendency. Much of it is due to the "vicious circle" which is set up. When much fat is deposited the digestion, which has probably been at fault from the beginning, becomes more faulty still, while the growing weight of the body makes the necessary exercise more and more difficult to obtain.

Speaking in somewhat general terms, Sir Lauder Brunton laid it down that the treatment of obesity naturally consists in limiting the food both in quantity and quality, and increasing the combustion of the body by accelerating the circulation. Where from any cause it is impossible to take exercise, massage, by which the circulation through the muscles may be increased threefold, is a great resource, but, as he very truly said, massage alone is not sufficient, and must only be looked upon as a preliminary to more active exercises, while even exercise, unaccompanied by proper restrictions as to diet, will not by itself bring down obesity. As to drugs, special reference was made to the use of thyroid extract. This substance increases tissue change, and will reduce fat considerably, but the evil effects, such as nervousness, tachycardia and tremor, which are produced by the continued use of thyroid must not be overlooked, and in most cases it is found to be far better to diminish obesity by attention to feeding and exercise than by the use of drugs.

In considering the various diets that have been recommended from time to time for the removal of obesity, one notes at once that they nearly all, to a large extent, depend for their efficacy upon the fact that the total amount of food permitted, as measured by the number of calories of energy contained in it, is considerably reduced. Thus, whether we take the Banting, Oertel, Ebstein, Hirschfeld, or Von Noorden diets, we find that the total energy contained in them is so reduced that the patients are forced to "live upon themselves" to a considerable extent. Even in the "beef-steak cure" a large proportion of the meat eaten is mere padding, so that it also has to be classed among the "hunger cures." As was pointed out by Dr. Schott, the treatment by a rigid and limited milk diet, and that by dry rolls, the amount of which one can insalivate in the course of a day is strictly limited, are also "hunger cures." This brings us to the question of withholding fluid. It seems to be well ascertained that in many cases the absolute avoidance of fluid at meal times is a very useful measure in diminishing obesity, but there is good ground for believing that this again operates partly by diminishing the amount of food taken. As Dr. Hutchison pointed out, the mere diminution of the total fluid taken has not been found experimentally to produce a diminution of weight, the reduction noted in Germany under such circumstances being probably due to the fact that the fluid which was reduced was beer. After all there is probably no one diet that will do for all cases of obesity, and one may safely say that while in nearly all cases the total diet must be reduced, and that as a general thing the fats and carbohydrates must be reduced more than the proteids, the art of adjusting the diet to the case lies chiefly in ascertaining whether the stress of the reduction can be most safely thrown upon the fats or the carbohydrates, always remembering that whenever the diet is made to consist largely of proteid elements a proportionate quantity of water must be given to remove the waste.

